# Serum concentration of zinc is elevated in clinically stable bipolar disorder patients

**DOI:** 10.1002/brb3.2472

**Published:** 2021-12-30

**Authors:** Bo H. Jonsson, Funda Orhan, Sanna Bruno, Ana Osório Oliveira, Timea Sparding, Mikael Landen, Carl M. Sellgren

**Affiliations:** ^1^ Department of Clinical Neuroscience, Centre for Psychiatry Research Stockholm & Stockholm Health Care Services Stockholm County Council Karolinska Institutet Stockholm Sweden; ^2^ Department of Physiology and Pharmacology Karolinska Institutet Stockholm Sweden; ^3^ Section of Psychiatry and Neurochemistry The Sahlgrenska Academy at University of Gothenburg Sahlgrenska University Hospital Gothenburg Sweden; ^4^ Department of Medical Epidemiology and Biostatistics Karolinska Institutet Stockholm Sweden

**Keywords:** bipolar patients, cognition, C‐reactive protein, serum, zinc

## Abstract

**Background:**

Bipolar disorder (BD) is a chronic psychiatric disorder characterized by recurrent mood episodes interspersed with euthymic periods. A growing number of studies have indicated that zinc plays an important role in coordinating immune responses, as well as being involved in synaptic transmission. In the current study, we set out to measure serum levels of zinc in a meticulously phenotyped cohort of 121 euthymic BD subjects and 30 matched controls.

**Methods:**

Serum levels of zinc were measured by photometry. To assess the interplay between zinc levels and immune activation in BD, we measured serum levels of high‐sensitive C‐reactive protein (hsCRP) levels by immunoturbidimetric assay, and serum levels of monocyte chemoattractant protein‐1 (MCP‐1), chitinase 3‐like protein 1 (YKL‐40), and soluble cluster of differentiation 14 (sCD14) by electrochemiluminescence enzyme‐linked immunosorbent assays. The baseline clinical diagnostic instrument for BD was the Affective Disorder Evaluation, and executive functioning was assessed by using the Delis–Kaplan Executive Function System.

**Results:**

Controlling for potential confounding factors, BD patients displayed increased serum levels of zinc unrelated to hsCRP, MCP‐1, YKL‐40, and sCD14 levels. Serum levels of zinc did not associate with executive functioning or measurements of disease severity.

**Discussion:**

This study suggests that the zinc homeostasis is disturbed in BD and that this dyshomeostasis is not related to ongoing mood symptoms or immune activation. Of note, serum levels were increased and hence do not support continuous zinc supplementation in BD.

## Graphical abstract

Clinically stable bipolar disorder patients, compared to healthy matched controls, displayed elevated serum zinc levels unrelated to immune activation. Serum levels of zinc did not associate with executive functioning or measurements of disease severity.

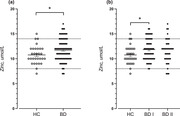

## 1 INTRODUCTION

Bipolar disorder (BD) is a chronic psychiatric disorder characterized by recurrent mood episodes interspersed by euthymic periods. The disorder has an early age of onset, and the majority of the affected subjects display their first symptoms in late adolescence or early adulthood (Nowrouzi et al., 2016). As a group, BD patients display deficits across multiple domains of cognitive functioning that also go beyond the recurrent mood episodes (Cullen et al., 2016; Demmo et al., 2018; Sparding et al., 2015). Such deficits, particularly those affecting executive functioning, have also been linked to worse functional outcomes (Burdick et al., 2010). BD is highly heritable, with twin studies reporting heritability estimates between 60% and 80% (Johansson et al., 2019). Genome‐wide association studies (GWAS) estimate that at least 30% of the heritability is due to common genetic variants, and to date, approximately 30 common genetic variants have been linked to BD (Stahl et al., 2019). These loci indicate genes such as *GRIN2A*, coding for the glutamate (NMDA) receptor subunit epsilon‐1 (NR2A), genes coding for ion channels and transporters (*RIMS1*, *ANK3*), and genes coding for zinc‐binding proteins (*ZCCHC2*). A large number of cross‐sectional studies (Benedetti et al., 2020; Rosenblat et al., 2014), and to some extent longitudinally designed studies (Isgren et al., 2017; Munkholm et al., 2018), have also reported promising immune‐related biomarkers for BD although differences between studies, such as stage of the disorder at sample collection, often have complicated interpretation.

Zinc is an essential micronutrient for all living organisms and is a crucial component of various enzymes and other proteins (Vallee & Falchuk, 1993). In fact, approximately 10% of proteins encoded in the human genome is potentially zinc binding (Andreini et al., 2006), reflecting the importance of this mineral in multifarious biological processes including cognitive function (Prasad, 2013). A growing number of studies have indicated that zinc plays an important role in coordinating immune responses (Hojyo & Fukada, 2016), as well as being involved in synaptic transmission (Frederickson et al., 2005). In the brain, zinc can foremost be found in a chelatable ionic form located in synaptic vesicles of glutamatergic neurons (Sensi et al., 2009; Vogt et al., 2000). The NR2A subunit of NMDARs then displays a sensitivity to extracellular zinc in the nanomolar range that mediates allosteric inhibition (Nozaki et al., 2011; Paoletti et al., 1997, 2000). Thus, a growing interest in zinc‐dependent signaling has emerged in the context of brain disorders (Frederickson et al., 2005). For example, free zinc in the extracellular compartment has been shown to induce amyloid deposition (Alzheimer's disease) (Bush et al., 1994; Frederickson et al., 2005), and abnormalities in zinc‐metalloproteins have been linked to amyotrophic lateral sclerosis (ALS) (Frederickson et al., 2005). It has also been proposed that low levels of zinc at the NMDAR contribute to depressive symptoms (Mlyniec, 2015), and clinical studies have reported decreased serum levels of zinc in subjects suffering from major depressive disorder (Swardfager et al., 2013). Further, in clinical trials, zinc supplementation has been reported to improve depressive symptoms (Yosaee et al., 2020). In BD, fewer studies and with limited sample sizes have been performed but reported decreased serum zinc levels in depressed bipolar patients, while euthymic patients displayed similar zinc levels as controls (Millett et al., 2017; Santa Cruz et al., 2020; Siwek et al., 2016).

In the current study, we set out to study serum zinc levels in a well‐characterized cohort of 121 clinically stable BD patients, not in an ongoing mood episode, and 30 matched healthy controls. To study associations with markers of immune activation in BD, we also measured serum levels of high‐sensitive C‐reactive protein (hsCRP) (Fernandes et al., 2016), monocyte chemoattractant protein‐1 (MCP‐1) (Misiak et al., 2020), chitinase 3‐like protein 1 (YKL‐40) (Jakobsson et al., 2015), and the soluble cluster of differentiation 14 (sCD14) (Jakobsson et al., 2015). Given the detailed clinical characterization, we could also study zinc levels in relation to clinical phenotypes, including executive functioning.

## 2 MATERIALS AND METHODS

The study was approved by the Regional Research Ethics Board in Stockholm and adhered to the tenets of the Declaration of Helsinki. All BD patients and healthy controls were enrolled, after providing verbal and written informed consent.

### 2.1 Study population

Patient data were collected from BD patients at a tertiary outpatient unit at the Northern psychiatric clinic in Stockholm, Sweden, as part of the St. Göran bipolar project, a Swedish prospective longitudinal study on the course of illness in BD. In the current study, all used data were collected at a 7‐year follow‐up. Details regarding the diagnostic procedure can be found in previous publications (Abé et al., 2020; Sellgren et al., 2019, 2016). Briefly, the diagnostic assessments were based on all available sources of information, including medical records and interviews with family members when feasible. A consensus panel of experienced board‐certified psychiatrists specialized in BD arrived at “best estimate” diagnoses. Enrolled study subjects were at least 18 years of age and met the Diagnostic and Statistical Manual of Mental Disorders criteria for a BD I, II, or not otherwise specified (NOS) diagnosis. A clinical assessment together with The Montgomery–Åsberg Depression Rating Scale (MADRS) (Montgomery & Åsberg, 1979) and the Young Mania Rating Scale (YMRS) (Young et al., 1978) were used to assess the extent of ongoing depressive and manic symptoms in patients. None of the included patients were deemed to be in an ongoing mood episode. The baseline clinical diagnostic instrument for BD was the Affective Disorder Evaluation (ADE) (Sachs et al., 2003), translated and modified to fit Swedish conditions after permission from the originator. Co‐morbid psychiatric disorders were collected using the Mini International Neuropsychiatric Interview (M.I.N.I.) (Sheehan et al., 1998). The Global Assessment of Functioning (GAF) (American Psychiatric Association, 2000) and the Clinical Global Impression scale (CGI) (Luborsky, 1975) were used to assess the functional impact and disease severity. The Drug Use Disorders Identification Test (DUDIT) (Berman et al., 2005) and Alcohol Use Disorders Identification Test (AUDIT) (Saunders et al., 1993) were used to screen for substance and alcohol abuse. None of the participants were on dietary zinc supplementation as per oral reporting and/or medical health records.

The general population healthy controls were randomly selected from the same catchment area by Statistics Sweden and underwent a similar clinical evaluation as the BD patients (Abé et al., 2020; Sellgren et al., 2019, 2016).

### 2.2 Blood collection and zinc analyses

Blood sampling was performed in the morning after an overnight fast and in conjunction with the collection of the clinical data. Peripheral blood was obtained in the morning using standard venipuncture techniques. Serum was isolated by coagulation and centrifugation. Measurement of serum zinc was carried out using a colorimetric method by photometry at 560 (550–580) nm with Advia 1800 Chemistry System (Siemens, Healthineers). The limit of detection was 0.612 μmol/L.

### 2.3 Analyses of hsCRP, MCP‐1, YKL‐40, and sCD14

Blood levels of hsCRP were analyzed using an immunoturbidimetric assay (Siemens, Healthineers). The limit of quantification was 3 mg/L. The analysis was carried out by a commercial laboratory (Unilabs AB, Stockholm, Sweden). MCP‐1 levels were analyzed using commercial electrochemiluminescence enzyme‐linked immunosorbent assays (ELISA; Human MCP‐1 Ultra‐Sensitive Kit), and sCD14 and YKL‐40 were analyzed using commercial colorimetric ELISAs (Human sCD14 quantikine ELISA kit, Human chitinase‐3 quantikine ELISA kit, R&D Systems Inc.) at the Clinical Neurochemistry Laboratory in Mölndal, Sweden. Intra‐assay coefficients of variation were below 10% for all assays. The staff performing the analyses were blinded, to patient identity and diagnosis (Jakobsson et al., 2015).

### 2.4 Neuropsychological testing

The neuropsychological assessments were performed on the same day as the blood collection and carried out by psychology students supervised by an experienced clinical psychologist. The Wechsler Adult Intelligence Scale III was used to assess general cognitive ability (Wechsler, 1997) as measured by the full‐scale intelligence quotient (IQ). Executive functioning was assessed using selected parts of the Delis–Kaplan Executive Function System (D‐KEFS) that consists of nine subtests used to evaluate key components of executive function such as mental flexibility, concept formation, problem‐solving, and inhibition. In the current study, we used scaled scores (adjusted for age and sex) from all conditions across three individual tests in D‐KEFS: (1) the Color‐Word Interference Test (CWIT), measuring the ability to inhibit automatic verbal responses, (2) The Verbal Fluency Test (VFT), providing information about language skills and verbal processing ability, as well as problem‐solving and inhibition, and (3) The Trail Making Test (TMT), measuring cognitive flexibility, visual attention, and motor speed.

### 2.5 Statistical analyses

All analyses were performed using R Statistics (version 4.0.3) or IBM SPSS (23.0) for Windows (IBM Inc.). Graphs were produced using Prism version 8.0 (GraphPad Software Inc.) or R Statistics. The normality of the data was determined using D'Agostino and Pearson omnibus normality test. Between‐group differences (BD vs. healthy controls) in demographic and clinical characteristics were evaluated with unpaired *t*‐tests or Chi‐square tests. Comparisons of serum zinc levels between groups were performed using unpaired *t*‐tests (BD vs. healthy controls) as well as by using a univariate analysis of variance (ANOVA; BD type I vs. BD type II vs. healthy controls) and followed up by logistic regression analyses to control for indicated confounding. For the PCA model (to create a single executive functioning score), we included a total of 52 subtests (due to missing data “TMT all error types number–letter switching condition 4,” “CWIT all error scores condition 2,” and “CWIT word reading and inhibition condition 3” were omitted). The first and second principal components (PC1 and PC2) were then used in a correlation analysis against serum zinc levels followed up by partial correlation analyses adjusting for IQ. The loadings of each test on PC1 were then also assessed and the three subtests displaying the highest loading on each PC were studied against zinc levels (then using the complete number of observations for that specific subtest, i.e., also included subjects with missing data for other subtests). As a sensitivity analysis, to exclude significant effects of potential outliers, we also performed a robust PCA analysis and used the first low‐rank component for correlation to zinc levels. Analyses were performed using the rpca package in R statistics. The assumptions of each statistical model were checked and reported *p*‐values are two sided.

## 3 RESULTS

### 3.1 Demographic and clinical characteristics

Demographic and clinical characteristics of the BD patients (*n* = 121) and healthy controls (*n* = 30) are presented in Table 1. Sixty‐nine patients met the DSM‐IV criteria for a BD type I diagnosis, 40 for a BD type II diagnosis, and 12 patients received a BD NOS diagnosis. All subjects were considered to be in a clinically stable phase, that is, not in an ongoing mood episode, at the time of data collection. The mean body mass index (BMI) was significantly higher in patients compared with controls, and none of the controls were smokers, while 23% of the patients were smokers.

**TABLE 1:** Demographic and clinical characteristics of the study population

	MEAN ± SEM (*n*)*		
Characteristics	Healthy controls (*n *= 30)	BD Patients (*n *= 121)	*p*‐value
Age (years)	46.37 ± 2.80 (30)	46.38 ± 1.25 (121)	.99^a^
Sex (male/female)	13/17	49/72	.84^b^
BMI (kg m^−2^)	24.31 ± 0.61 (30)	26.36 ± 0.44 (121)	**.03** ^a^
% Smokers	0%	23%	**.001** ^b^
Medication:			
Lithium	0%	58%	—
Antipsychotics	0%	28%	—
Antidepressants	0%	38%	—
Sedatives	0%	32%	—
Anti‐epileptics	0%	26%	—
Contraceptives or HRT	23.5%	8.3%	.10
Diagnosis (DSM IV)			
BD type I	—	57% (69)	—
BD type II	—	33% (40)	—
BD NOS	—	10% (12)	—
Symptoms ratings			
MADRS total	1.46 ± 0.51 (30)	3.83 ± 0.40 (118)	**.005** ^1^
GAF disability	—	63.69 ± 0.97 (105)	—
YMRS	0.50 ± 0.26 (30)	1.10 ± 0.17 (115)	.06
GAF symptom	—	64.92 ± 0.86 (105)	
CGI‐bipolar improvement	—	4.70 ± 0.12 (104)	—
CGI‐bipolar depression	—	1.50 ± 0.09 (98)	—
CGI‐bipolar mania	—	1.13 ± 0.049 (98)	—
CGI total	—	3.87 ± 0.085 (104)	—

*Abbreviations*: BD, Bipolar disorder; BMI, body mass index; CGI, clinical global impression; CRP, c‐reactive protein; GAF, global assessment of functioning; HRT, hormone replacement therapy; MADRS, Montgomery–Åsberg Depression Rating Scale; NOS, not otherwise specified; YMRS, Young Mania Rating Scale.

* Unless otherwise indicated.

^a^ Unpaired *t*‐test with equal SD.

^b^ Chi‐square test.

### 3.2 Increased serum levels of zinc in euthymic bipolar disorder

**FIGURE 1:**
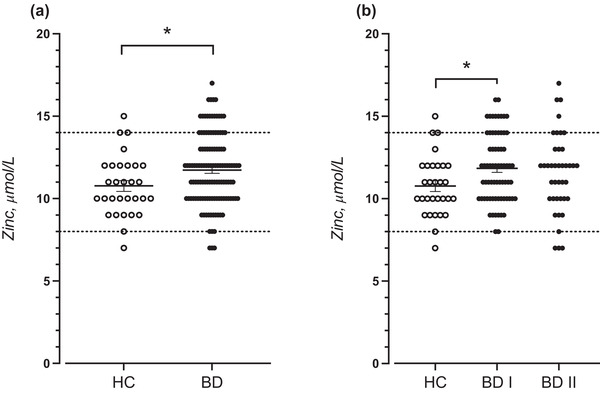
Serum zinc levels in (a) all bipolar disorder patients (BD) and (b) BD I and BD II patients compared with healthy controls (HC). Each data point represents the concentration of a single serum sample in units of μmol/L. Data are shown as mean ± SEM. Data were analyzed using an unpaired *t*‐tests with equal SD (a); *p* = .026, or a one‐way ANOVA with Tukey post hoc test (b); *p* = .035. *p < .05. Both *p*‐values are two sided. Reference interval for zinc concentration was 8–14 μmol/L

The serum levels of zinc (Figure 1a) were significantly elevated in BD patients compared with healthy controls (11.73 ± 0.20 μmol/L, *n* = 121 vs.10.77 ± 0.33 μmol/L, *n* = 30, respectively; *p* = .026). As BD patients displayed a higher mean BMI than healthy controls and were more commonly smokers, we also performed a binary logistic regression analysis adjusting for these factors. In line with the unadjusted analysis, the risk of being a patient significantly increased by increasing serum zinc levels (odds ratio = 1.28; *p* = .041). No significant correlations between serum zinc and BMI, age, sex, smoking, use of contraceptives, was either observed in BD patients (Table S1) or healthy controls (Table S2). In serum from BD patients, we observed no significant associations between levels of zinc and hsCRP, YKL‐40, MCP‐1, and sCD14 (Table S3). Serum zinc levels did not differ between BD patients that were on lithium treatment versus all non‐lithium treated patients (11.71 ± 0.27 μmol/L, *n* = 70, and 11.75 ± 0.29 μmol/L, *n* = 51, respectively; *p* = .94). Neither did serum zinc levels differ between treated and untreated BD patients with regards to antipsychotics (*p* = .47), antidepressants (*p* = .25), sedatives (*p* = .51), or anti‐epileptics (*p* = .13) Table S4). Finally, when stratifying by BD subtype, BD I subjects displayed significantly increased serum zinc levels as compared to healthy controls (11.84 ± 0.25 μmol/L, *n* = 69 vs.10.77 ± 0.33 μmol/L, *n* = 30, respectively; *p* = .040) (Figure 1a). Serum zinc levels did not differ between BD II subjects and healthy controls (11.55 ± 0.38 μmol/L, *n* = 40 vs.10.77 ± 0.33 μmol/L, *n* = 30, respectively; *p* = .21) (Figure 1b).

### 3.3 Serum levels of zinc do not associate with symptom severity in euthymic bipolar disorder

Serum levels of zinc did not significantly correlate to rated subsyndromal mood symptoms as measured by MADRS (*r* = −0.0921, *p* = .32), social, occupational, and psychological functioning as measured by GAF (GAF Symptom: *r* = 0.051, *p* = .60, GAF disability: −0.005, *p* = .96), general symptom severity (CGI‐bipolar depression: *r* = −0.062, *p* = .54, CGI‐bipolar Mania: *r* = −0.001, *p *= .99), or improvement over time (CGI‐bipolar improvement: *r* = 0.009, *p* = .93). We also performed analyses stratifying bipolar cases depending on type 1 or type 2 disorder, but without evidence of significant associations in the stratified groups. See also Table 2.

### 3.4 Serum levels of zinc do not associate with executive functioning in euthymic bipolar disorder

**FIGURE 2:**
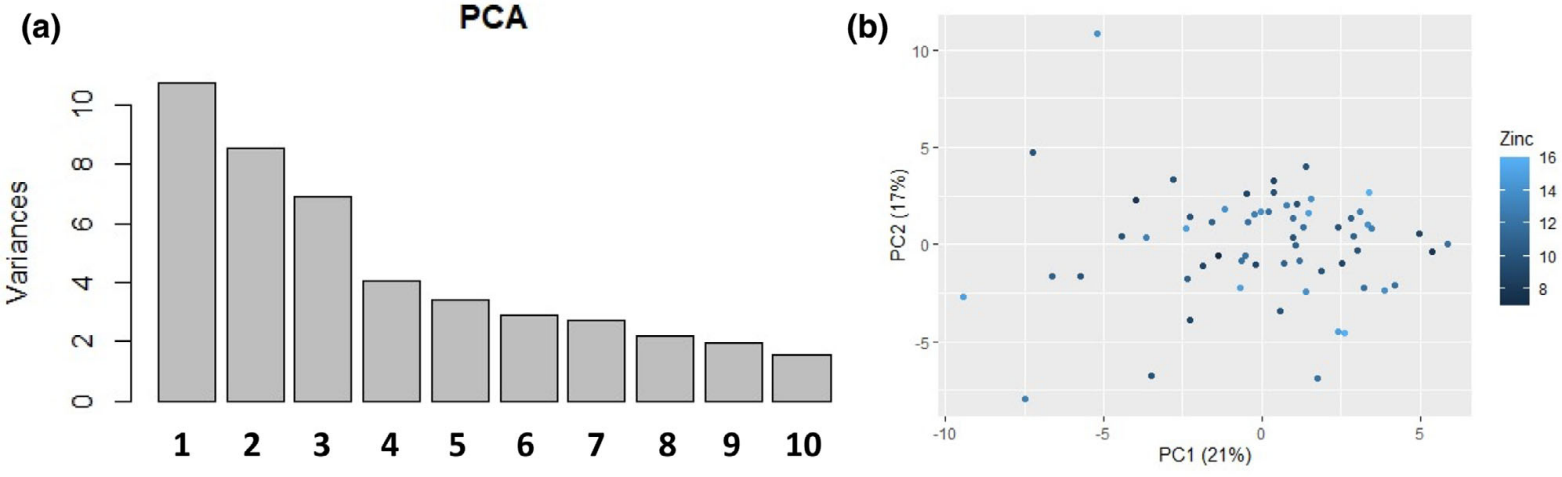
(a) Variance explained (percentages) for the first 10 principal component in the principal component analysis (PCA) using scaled scores of performance in 52 subtests of the Delis–Kaplan Executive Function System (D‐KEFS) from 60 euthymic bipolar disorder subjects. (b) Plot displaying loading on first (PC1) and second (PC2) principal component for 60 euthymic bipolar disorder subjects. Shades of blur represent serum zinc levels and the principal component analysis used scaled scores of performance in 52 subtests of D‐KEFS

With our selection of subtests from D‐KEFS (CWIT, VFT, and TMT), 60 patients had complete data, and this data was used to create a PCA model. PC1 accounted for 21% of the variance, while PC2 explained 17% of the variance (Figure 2). To evaluate the influence of zinc levels on executive functioning, we then performed correlation analysis with zinc levels as the independent variable and PC1 and PC2 as dependent variables. None of the correlation analysis suggested a significant correlation, either unadjusted for IQ (*r *= 0.07; *p* = .62, and *r* = 0.02; *p* = .90, respectively), or adjusted for IQ (*r* = 0.15; *p* = .26, and *r* = 0.02; *p* = .87, respectively), see also Table 3 and Figure 2. For CWIT, “condition 4 inhibition/switching” displayed the highest loading on PC1 (0.25), while for VFT the “second interval” displayed the highest loading (0.22), and for TMT “condition 4 number + letter switching” displayed the highest loading (0.20). For PC2, For CWIT, “inhibition/Switching vs. inhibition” displayed the highest loading on PC1 (0.09), while for VFT “percent repetition errors” displayed the highest loading (0.09), and for TMT “switching vs. combined number + letter sequencing” displayed the highest loading (0.30). Each of these subtests was also studied separately against zinc levels but then also included subjects with missing data on other subtests and hence not included in the PCA model. However, no significant associations could be observed (Table 3). Finally, to exclude that outliers significantly influenced our PCA model, and the correlations between our composite cognitive scores and zinc levels, we also performed a robust PCA model and used the first low‐rank component for correlation to zinc levels but without evidence of a significant association (*r* = −0.15; *p* = .23).

**TABLE 2:** Executive functioning and serum levels of zinc in euthymic bipolar disorder patients

		Correlation against serum levels of zinc		
Neurocognitive test		*r*	*p*‐value	*n*
Principal component 1 (PC1)		0.06	.65	62
PC1 (adjusted for IQ)		−0.08	.51	62
Principal Component 2 (PC2)		−0.05	.71	62
PC2 (adjusted for IQ)		−0.08	.52	62
	Loading on PC1			
CWIT, condition 3 inhibition	0.22	0.20	.10	65
CWIT, condition 3 inhibition (adjusted for IQ)		−0.08	.51	65
VFT, category fluency, total correct	0.22	0.00	.98	68
VFT, the second interval (adjusted for IQ)		−0.09	.49	68
TMT combined number + letter sequencing	0.25	−0.15	.23	61
TMT condition 4 number + letter switching (adjusted for IQ)		−0.11	.42	61
	Loading on PC2			
CWIT inhibition/switching vs. combined naming + reading	0.18	0.12	.34	65
CWIT inhibition/switching vs. inhibition (adjusted for IQ)		−0.08	.55	65
VFT Letter fluency, Total correct	0.14	0.05	.68	68
VFT percent repetition errors (adjusted for IQ)		−0.08	.49	68
TMT switching vs. visual scanning	0.27	0.13	.34	57
TMT switching vs. combined number + letter sequencing (adjusted for IQ)		−0.09	.48	57

**TABLE 3:** Symptom ratings and their association with serum zinc in bipolar disorder

	All BD patients		BD type I patients		BD type II patients	
Symptom ratings	*r*	*p*‐value^a^	*r*	*p*‐value^a^	*r*	*p*‐value^a^
MADRS total	−0.16	.090	−0.16	.20	−0.18	.27
GAF disability	−0.026	.80	−0.0033	.98	−0.027	.87
GAF symptom	0.066	.52	0.060	.66	0.12	.48
CGI bipolar improvement	0.057	.58	0.12	.37	0.019	.91
CGI depression	−0.11	.32	−0.24	.088	0.072	.67
CGI mania	−0.075	.48	−0.16	.24	0.016	.92

*Abbreviations*: BD, bipolar disorder; CGI, clinical global impression; GAF, global assessment of functioning; MADRS, Montgomery–Åsberg Depression Rating Scale.

^a^ Spearman correlation.

## 4 DISCUSSION

In the current study, we measured serum concentrations of zinc in a well‐defined group of 121 clinically stable BD patients and made comparisons with healthy controls randomly selected from the normal population. Adjusting for potential confounders, we observed higher serum zinc concentrations in BD patients, not in an ongoing mood episode, than in controls, with 13% of BD cases displaying zinc concentrations above the reference interval (as compared to 3% in healthy controls). Using data from a detailed clinical profiling, we observed no associations between zinc concentrations and disease severity or executive functioning. So far, meta‐analyses of clinical studies have not either been able to show a significant effect of zinc supplementation on cognitive functioning in healthy populations (Warthon‐Medina et al., 2015).

In 2007, Nourmohammadi et al. (2007) described lower serum zinc concentrations in 30 bipolar patients. However, those patients were sampled across different mood phases. In 2011, González‐Estecha et al. (2011) observed higher serum concentrations of zinc in 25 BD patients. Their study population consisted of both patients in a manic as well as in a depressed phase, and follow‐up analyses indicated the highest zinc concentrations in manic patients. In 2016, Siwek et al. (2016) then observed lower serum zinc concentrations in 58 depressed BD type 1 patients, that is, the same subgroup that here displayed increased zinc levels although in an euthymic phase, while another 48 BD patients in remission displayed zinc concentrations in line with healthy controls. Further, zinc concentration did not correlate to depression severity. Analyses were adjusted for age, sex, duration of illness, as well as a number of previous episodes. In 2017, Millett et al. (2017) reported lower serum zinc concentrations in 27 bipolar patients as compared to healthy controls. These patients were also in different mood phases. Recently, Santa Cruz et al. (2020) then reported lower serum concentrations of zinc in 15 BD patients indicated to be in remission according to ratings of ongoing depressive and manic symptoms.

Although serum concentrations of zinc only reflect a small proportion of the human body zinc pool, obfuscating interpretation of individual assessments, measuring the serum concentration of zinc is usually the recommended method to assess zinc status at the population level, especially if conducted under standardized conditions and preferably collected as morning fasting samples. Several factors such as the use of contraceptives, pregnancy, albumin levels, smoking, ongoing infection, and food have then been shown to influence the serum concentration of zinc and need to be taken into account. In the current study, such data were also collected and could not explain the higher concentration observed in BD patients.

The lack of associations between markers of immune activation and zinc levels suggests that functional implications in BDs may be more related to enzyme activities and synaptic transmission. Notably, several BD loci involve zinc‐binding proteins (Stahl et al., 2019) and NMDARs display a sensitivity to extracellular zinc even in the nanomolar range (Nozaki et al., 2011; Paoletti et al., 1997, 2000). However, to what extent the observed increase then reflects a disturbed homeostasis in the brain remains elusive and cannot be extrapolated from the current data. Given the difficulties in assessing the zinc concentration in vivo at the synapse, a reasonable next step would be to study the influence of selected genetic risk variants on serum zinc concentrations in BD.

The current study only included patients that were not in an ongoing mood episode. While this enabled us to study zinc levels in a large cohort of bipolar patients within a similar mood state, it limited us from studying how zinc levels potentially vary across mood episodes (although no correlations were observed between zinc levels and subsyndromal mood symptoms). As previous studies, although with limited sample sizes, suggest decreased levels in depressed bipolar patients it would be of interest to perform prospective investigations measuring serum zinc during different mood levels in the same patients. Importantly, such studies then also need to adjust for time‐dependent factors potentially altering the distribution of zinc within the complete pool.

In sum, this study indicates that zinc homeostasis is disturbed in BD as reflected by increased serum zinc concentrations in clinically stable BD patients. To what extent the increased serum levels in remitted patients have implementations for brain pathophysiology, or to what extent they reflect genetic risk factors, remains to be investigated.

## CONFLICT OF INTEREST

Carl M. Sellgren is a scientific adviser for Outermost Therapeutics (of no relevance to this work). The authors have no financial conflicts of interest.

## AUTHOR CONTRIBUTIONS

Bo H. Jonsson, Funda Orhan, Mikael Landen, and Carl M. Sellgren conceived and designed the study. Carl M. Sellgren, Mikael Landen, and Bo H. Jonsson collected the clinical data. Timea Sparding supervised the collection of the cognitive data. Bo H. Jonsson, Funda Orhan, Sanna Bruno, and Ana Osório Oliveira analyzed the data. Carl M. Sellgren supervised the project. Bo H. Jonsson, Funda Orhan, and Carl M. Sellgren wrote the manuscript and all authors contributed with feedback.

### PEER REVIEW

The peer review history for this article is available at https://publons.com/publon/10.1002/brb3.2472

## Supporting information

Supporting information

## Data Availability

The datasets generated and analyzed during the current study are available from the corresponding author on reasonable request.
