# Supplementary material for: Serum concentration of zinc is elevated in clinically stable bipolar disorder patients
Source: Brain Behav. 2021 Dec 30;12(1):e2472. doi: 10.1002/brb3.2472 (PMC8785612; doi:10.1002/brb3.2472)
Supplement: Supplementary file 1 — Supporting information [file BRB3-12-e2472-s001.docx]

**Supplementary tables**

Table S1. Clinical characteristics and their association with serum zinc in bipolar disorder.

| Characteristics | r | P-value^1^ |
| --- | --- | --- |
| Age (years) | -0.026 | 0.78 |
| Sex (male/female) | -0.097 | 0.29 |
| BMI (kg m^-2^) | 0.15 | 0.11 |
| Smoking | -0.082 | 0.37 |
| hsCRP | -0.075 | 0.49 |
| Contraceptives or HRT | 0.047 | 0.70 |
| ^1^Pearson Correlation  Abbreviations: BMI = Body Mass Index, hsCRP = high-sensitive c-reactive protein, HRT = Hormone replacement therapy | | |

Table S2. Clinical characteristics and their association with serum zinc in healthy controls.

| Characteristics | r | P-value^1^ |
| --- | --- | --- |
| Age (years) | -0.26 | 0.17 |
| Sex (male/female) | -0.27 | 0.16 |
| BMI (kg m^-2^) | 0.30 | 0.10 |
| Smoking | N/A | N/A |
| hsCRP | -0.16 | 0.58 |
| Contraceptives or HRT | -0.22 | 0.40 |
| ^1^Pearson Correlation  Abbreviations: BMI = Body Mass Index, hsCRP = high-sensitive c-reactive protein, HRT = Hormone replacement therapy, N/A = not applicable | | |

**Table S3**. Immune markers and their association with serum zinc in bipolar disorder.

| Characteristics | r | P-value^1^ |
| --- | --- | --- |
| hsCRP | -0.075 | 0.49 |
| YKL-40 | -0.14 | 0.16 |
| MCP-1 | -0.055 | 0.58 |
| sCD14 | 0.084 | 0.39 |
| ^1^Pearson Correlation  Abbreviations: hsCRP = high-sensitive c-reactive protein, YKL-40 = chitinase 3-like protein 1, MCP-1 = monocyte chemoattractant protein-1,  sCD14 = soluble cluster of differentiation 14 | | |

**Table S4.** Serum zinc levels (µmol/L) with regard to medication in bipolar disorder.

|  | Patient on drug | Patient off drug |  |
| --- | --- | --- | --- |
| Medication | Serum zinc  MEAN ± SEM (n) | Serum zinc  MEAN ± SEM (n) | P-value^1^ |
| Lithium | 11.71 ± 0.27 (70) | 11.75 ± 0.29 (51) | 0.94 |
| Antipsychotics^2^ | 11.50 ± 0.36 (34) | 11.82 ± 0.23 (87) | 0.47 |
| Antidepressants^3^ | 11.43 ± 0.30 (46) | 11.91 ± 0.26 (75) | 0.25 |
| Sedatives^4^ | 11.54 ± 0.33 (39) | 11.82 ± 0.24 (82) | 0.51 |
| Anti-epileptics^5^ | 12.22 ± 0.40 (32) | 11.55 ± 0.22 (89) | 0.13 |
| ^1^Unpaired t-test with equal SD  ^2^All antipsychotics combined  ^3^All antidepressants combined  ^4^All sedatives combined  ^5^All anti-epileptics combined | | | |
